# Associations of *Helicobacter pylori* infection and peptic disease with diabetic mellitus: Results from a large population-based study

**DOI:** 10.1371/journal.pone.0183687

**Published:** 2017-08-29

**Authors:** Saeda Haj, Gabriel Chodick, Rotem Refaeli, Sophy Goren, Varda Shalev, Khitam Muhsen

**Affiliations:** 1 Department of Epidemiology and Preventive Medicine, School of Public Health, Sackler Faculty of Medicine, Tel Aviv University, Tel Aviv, Israel; 2 Medical division, Maccabi Health Services, Tel Aviv, Israel; University of the Pacific, UNITED STATES

## Abstract

**Background:**

Evidence is conflicting regarding the association between *Helicobacter pylori* infection and diabetes mellitus. The study objective was to examine associations of *H*. *pylori* infection, gastric ulcers and duodenal ulcers, with diabetes mellitus.

**Methods:**

This cross-sectional study was undertaken using coded data from the computerized database of Maccabi Health Services in Israel, on 147,936 individuals aged 25–95 years who underwent the urea breath test during 2002–2012. Multiple logistic regression models were conducted, while adjusting for known risk factors for diabetes mellitus.

**Results:**

A *H*. *pylori* positive test was recorded for 76,992 (52.0%) individuals and diabetes for 12,207 (8.3%). The prevalence of diabetes was similar in individuals with and without *H*. *pylori* infection, but this association was modified (P for heterogeneity 0.049) by body mass index (BMI): adjusted odds ratio (aOR) 1.16 (95% confidence intervals (CI) 1.04–1.29) in persons with BMI<25 kg/m^2^ versus aOR 1.03 (95% CI 0.98–1.08) in persons with BMI≥25 kg/m^2^. Diabetes mellitus prevalence was higher in persons with gastric (aOR 1.20 (95% CI 1.06–1.34)) and duodenal ulcers (aOR 1.20 (95% CI 1.12–1.28)) compared to persons without these diagnoses.

**Conclusions:**

In this large population-based study, we demonstrated significant positive associations, albeit of small magnitude, of *H*. *pylori* infection and peptic disease with diabetes. The long-term gastric inflammation and associated-damage to the gastric mucosa might play a role in such associations.

## Introduction

Diabetes mellitus (DM) is a major public health problem, with increasing prevalence globally [1]. The major burden (90–95%) is caused by type 2 DM, which typically develops in adulthood and is characterized by variable levels of insulin resistance, impaired insulin secretion and increased glucose production [2]. Established risk factors for type 2 DM include sociodemographic factors [3–5] and lifestyle e.g., obesity, physical inactivity, poor diet and smoking [3, 6, 7]. However, these factors do not fully explain the occurrence of the disease.

Infections have been postulated to play a role in the pathogenesis of type 2 DM [8, 9], through the involvement of low grade systemic inflammation. The bacterium *Helicobacter pylori* is of particular interest, given its chronic course and persistent inflammation.

*H*. *pylori* colonizes the human stomach for decades, usually without causing apparent disease. *H*. *pylori* causes chronic gastritis, gastric and duodenal ulcers, and in rare occasions gastric cancer and lymphoma, which usually develop during adulthood [10]. *H*. *pylori* infection might also play a role in extragastric diseases [11], such as unexplained iron deficiency anemia and idiopathic thrombocytopenia [12].

Several studies have addressed the relationship between *H*. *pylori* infection and DM; however, the findings are conflicting [8, 9, 13–17]. Two meta-analyses of observational studies reported significant positive associations between *H*. *pylori* infection and DM with pooled odds ratios of 1.33 and 2.0 [18, 19]. However, the crude results of the original studies were combined in these meta-analyses, rather than the adjusted results; therefore, the impact of confounding could not be ruled out. Consideration of age, socioeconomic status and body mass index (BMI) are especially important, given the associations of these factors with both *H*. *pylori* infection and DM [4–7, 20, 21]. Adjustment for these and other confounders was limited in many of the previous studies on *H*. *pylori* and DM. Moreover, most of the evidence on the association between *H*. *pylori* infection and DM is based on small samples. Large population-based studies are essential, since the postulated relationship between *H*. *pylori* infection and DM appears to be of small magnitude. Gastric inflammation and damage to the gastric mucosa may be at the base of an association between *H*. *pylori* infection and DM. However, the role of peptic ulcers, which develop following persistent gastric inflammation in DM, has rarely been assessed.

The aim of the current large population-based study was to examine associations of *H*. *pylori* infection, and of gastric and duodenal ulcers, with DM. We also examined whether such associations differ by age and BMI. Our hypothesis was that positive associations would be found of *H*. *pylori* infection, and of related gastroduodenal diseases, with DM prevalence.

## Materials and methods

### Study design and population

A cross-sectional study was conducted using anonymous data retrieved from the computerized database of Maccabi Health Services (MHS). MHS is the second largest health maintenance organization in Israel, with two million insured persons (~25% of the Israeli population). The source population comprised adults aged ≥25 years insured in MHS who performed the Urea Breath Test (UBT) between 2002 and 2012. Individuals were excluded from the study population if they used anti-*H*. *pylori* eradication therapy (according to purchases of medications) or proton pump inhibitors four weeks before the UBT. Persons with a history of bariatric surgery and recent cancer diagnosis (within three years from the UBT) were also excluded.

### Data extraction and definitions

Information was obtained on the results of UBT, diagnoses of DM, hypertension, dyslipidemia, gastric ulcers, duodenal ulcers, *H*. *pylori*-gastritis, birth year, sex, town of residence, smoking (ever, never, and unknown) and BMI (weight in kilograms (kg)/height^2^ in meters (m)).

*H*. *pylori* positivity was defined based on the UBT result; individuals were classified as *H*. *pylori* positive if they had a UBT result >3.5, and negative if the test result was ≤3.5. If more than one UBT was performed, we used the first test to determine *H*. *pylori* positivity. The presence of gastroduodenal diseases was defined based on the following codes of the international classification of disease, 9^th^ revision (ICD-9): *H*. *pylori*-gastritis (41.86), gastric ulcer (531) and duodenal ulcer (532).

DM was defined based on entry to the MHS DM registry. Persons having at least one of the following criteria were classified as diabetic: 1) HbA1c ≥7.25%; 2) glucose ≥200 mg/dL; 3) diagnosis in the medical chart of DM based on any ICD-9 relevant code, and HbA1c ≥6.5% or glucose>125 mg/dL; 4) purchase of anti-diabetic medications twice in the previous two months [22]. Anti-diabetic medications can be purchased by prescription only, and data are routinely sent to primary care physicians for final validation.

Hypertension was defined based on entry to the MHS hypertension registry [23]. Individuals were classified as hypertensive if they had two or more physician's diagnoses of hypertension or diagnosis from hospital records, and two or more blood pressure measurements with systolic blood pressure ≥140 mmHg or diastolic blood pressure ≥90 mmHg. If a diagnosis of hypertension was lacking, four documented abnormal blood pressure measurements were required with ≥50% of systolic and diastolic blood pressure measurements of >160 and >90 mmHg, respectively. Persons with ≥6 dispensed hypertension medications were also classified as hypertensive [23].

Dyslipidemia was determined based on diagnoses codes (ICD-9^th^ code 272 and its subhierarchy, and equivalent MHS internal codes), documented by a physician twice or more in the medical record.

Socioeconomic status was defined based on the socioeconomic rank of place of residence at the level of town, as defined by the Israel Central Bureau of Statistics [24]. The ranks are on a scale from 1 to 10, with higher ranks representing a higher socioeconomic status. This aggregative socioeconomic index reflects a combination of basic characteristics of a specific geographical unit investigated, mainly financial resources of the residents, housing conditions, motorization level, education and employment [24]. Communities with socioeconomic ranks of 1–5, 6–7 and 8–10 were classified as low, intermediate and high, respectively. Country of birth was classified as: Israel, Former Soviet Union (FSU), America/Europe (excluding countries of the FSU), North Africa/Asia and other/unknown.

### Statistical analysis

The Chi square test was used to examine associations of DM with *H*. *pylori* infection and with related gastroduodenal diseases. The Chi square test was also used to examine differences between *H*. *pylori* infected and uninfected individuals in sociodemographic variables, BMI and smoking; as well as differences between diabetic and non-diabetic patients according to these variables. Age-adjusted Mantel-Haenszel Odds Ratios (OR_MH_) and the corresponding 95% confidence intervals (CIs) were calculated to examine associations of *H*. *pylori* infection (based on UBT), gastric ulcer and duodenal ulcer, with DM prevalence.

Multivariable logistic regression models were fitted, while adjusting for age, socioeconomic status, country of birth, hypertension, dyslipidemia, BMI and smoking. Adjusted OR (aOR) and 95% CIs were obtained from these models. The analyses were performed in stratification by age group (25–29, 30–39, 40–49, 50–59, 60–69 and 70–95 years) and BMI (<25 versus ≥25 kg/m^2^), separately for each exposure variable: *H*. *pylori* infection (based on UBT), gastric ulcer, and duodenal ulcer. Additional analysis was done while adding both variables *H*. *pylori* infection (based on UBT) and gastric ulcer, as well as a different analysis with the variables *H*. *pylori* infection and duodenal ulcer.

Using the chi square test for heterogeneity, the effect modification by age and BMI was assessed.

Two-sided P<0.05 was considered significant.

Data were analysed using SPSS version 23 (IBM, Armonk, New York, USA) and WinPepi [25]

### Ethical consideration

The study protocol was approved by the Helsinki committee of Assuta Medical Center and the ethics committee of Tel Aviv University. Since this is a retrospective study in which we used coded (anonymized) administrative data from electronic medical records, exemption from informed consent was granted by the Helsinki committee.

## Results

Among 158,059 individuals aged 25–95 years who underwent UBT between 2002 and 2012, 147,936 met study inclusion criteria and their data were analyzed. The mean age of the eligible persons was 42.8 years (standard deviation 12.7); about 39% of them were males, and 24.3% were born in the FSU. In total, 76,965 (52.0%) were *H*. *pylori* positive (UBT>3.5). Diagnoses of *H*. *pylori*-gastritis, gastric ulcer and duodenal ulcer were documented in 50,035 (33.8%), 3153 (2.1%) and 10,366 (7.0%) individuals, respectively. DM was determined in 12,2207 (8.3%) persons and obesity (BMI≥30 kg/m^2^) in 25,910 (17.5%).

The prevalence of *H*. *pylori* infection (based on UBT results) increased from 48.2% in persons aged 25–29 years to 55.1% and 56.9% in persons aged 30–39 and 40–49 years, respectively; and decreased gradually in older age groups. The prevalence of *H*. *pylori* infection differed according to country of birth, being highest in those born in the FSU countries (59%), and lowest (36%) in those born in the Americas/Europe (Table 1).

**Table 1:** *H*. *pylori* infection prevalence according to sociodemographic characteristics.

	Total	*H*. *pylori* positive, N (%)
**Age (years)**		
25–29	22,547	10,866 (48.2%)
30–39	46,357	25,542 (55.1%)
40–49	39,473	22,452 (56.9%)
50–59	22,014	10,737 (48.8%)
60–69	12,116	5274 (43.5%)
70–95	5429	2094 (38.6%)
**Sex, males**		
Men	58,173	30,787 (52.9%)
Women	89,763	46,178 (51.4%)
**Socioeconomic status rank** ^a^		
Low (1–5)	60,282	35,652 (59.1%)
Intermediate (6–7)	39,500	19,672 (49.8%)
High (8–10)	38,790	17,113 (44.1%)
Missing	9364	4528 (48.4%)
**Country of birth**		
Israel	96,180	48,831 (50.8%)
Former Soviet Union countries	35,885	21,024 (58.6%)
Asia/ North Africa	5379	2955 (54.9%)
Europe^b^ / America	7009	2550 (36.4%)
Other/unknown	3483	1605 (46.1%)
**BMI (kg/m**^**2**^**)** ^c^		
<18.5	4139	1879 (45.4%)
18.5–24	62,053	31,131 (50.2%)
25–29	49,636	26,301 (53.0%)
≥30	25,910	14,261 (55.0%)
Missing	6198	3393 (54.7%)
**Smoking**		
Ever	20,913	11,950 (57.1%)
Never	86,540	44,069 (50.9%)
Unknown	40,483	20,946 (51.7%)
**Hypertension**	33,317	16,426 (49.3%)
No hypertension	114,619	60,539 (52.8%)
**Dyslipidemia**	46,637	22,905 (49.1%)
No dyslipidemia	101,299	54,060 (53.4%)

^a^ Socioeconomic status(SES) rank of city/town of residence; 1–5 represents low SES; 6–7, intermediate SES; and 8–10, high SES.

^b^ Excluding countries of the Former Soviet Union.

^c^ BMI: body mass index; kg: kilogram; m: meter

**Fig 1:**
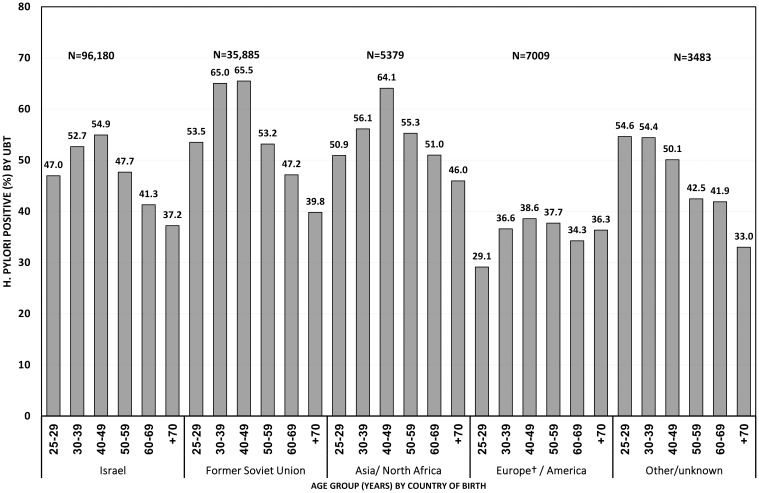
Prevalence of *H*. *pylori* infection (%) (urea breath test>3.5) by age group (in years) and country of birth; Maccabi Health Services, Israel 2002–2012. † Excluding countries that belonged to the Former Soviet Union.

The pattern of the age-specific prevalence of *H*. *pylori* infection found in the entire cohort (Table 1) was also evident for subgroups of persons who were born in Israel, in the FSU and in Asia/North Africa, but not for the subgroup of persons who were born in Europe and the Americas (Fig 1).

The prevalences of *H*. *pylori* infection was similar between men and women. A significant gradual decrease (P<0.001) was observed in the prevalence of *H*. *pylori* infection according to socioeconomic status of place of residence, and a significant increase was found with increasing BMI (Table 1). *H*. *pylori* infection according to UBT was associated with an increased likelihood of having a diagnosis code of *H*. *pylori*-gastritis (age-adjusted Mantel-Haenszel OR 3.86 (95% CI 3.76–3.94) and gastric ulcer (age-adjusted Mantel-Haenszel OR 1.20 (95% CI 1.11–1.28)).

The prevalence of DM increased significantly (P<0.001) with increased age and BMI, and decreased according to socioeconomic rank of place of residence (P<0.001). It also differed according to country of birth (S1 Table). DM was slightly more common in men than in women (P<0.001), and markedly more prevalent in individuals with hypertension and/or dyslipidemia (S1 Table).

### Associations of *H*. *pylori* infection, and of gastric and duodenal ulcer, with DM

Overall, in a crude analysis, prevalences of DM among *H*. *pylori* infected and uninfected patients were 7.9% vs 8.6%. Higher prevalence was found in persons with a diagnosis of gastric ulcer (14.8% vs 7.6%) and duodenal ulcer (14.1% vs 8.1%) than in those without such diagnoses.

#### Age-adjusted and age-stratified results

The age-stratified prevalences of DM according to each independent variable (*H*. *pylori* infection by UBT, gastric ulcer and duodenal ulcer), and BMI, are presented in Table 2.

**Table 2:** Prevalence of diabetes mellitus according to *H*. *pylori* infection, and gastric and duodenal ulcers, by age group and BMI.

	Age 25–29 years		Age 30–39 years		Age 40–49 years		Age 50–59 years		Age 60–69 years		Age 70–95 years	
**A: all BMI categories**	Total	DM (%)	Total	DM (%)	Total	DM (%)	Total	DM (%)	Total	DM (%)	Total	DM (%)
*H*. *pylori* positive	10,866	0.7	25,542	2.2	22,452	6.8	10,737	17.5	5274	26.8	2094	30.8
*H*. *pylori* negative	11,681	0.5	20,815	1.8	17,021	6.2	11,277	15.8	6842	25.9	3335	31.9
Gastric ulcer	295	0.3	678	2.9	783	8.7	680	21.3	448	32.1	269	33.1
No gastric ulcer	22,252	0.6	45,679	2.0	38,690	6.5	21,334	16.5	11,668	26.1	5160	31.4
Duodenal ulcer	775	1.4	2308	2.9	2637	8.3	2405	19.5	1458	29.6	783	31.5
No duodenal ulcer	21,772	0.6	44,049	2.0	36,836	6.4	19,609	16.3	10,658	25.8	4646	31.1
**B: BMI<25 kg/m**^**2**^												
*H*. *pylori* positive	6524	0.2	12,417	0.7	8910	0.2	3256	6.6	1350	14.1	553	19.3
*H*. *pylori* negative	7756	0.2	11,226	0.6	7583	1.5	3804	5.1	1934	13.3	879	20.0
Gastric ulcer	177	0.0	330	0.6	335	2.1	197	5.6	126	17.5	71	18.3
No gastric ulcer	14,103	0.2	23,313	0.6	16,158	1.8	6863	5.8	3158	13.5	1361	19.8
Duodenal ulcer	427	0.7	1021	0.7	968	4.0	705	7.2	385	15.3	193	21.2
No duodenal ulcer	13,853	0.2	22,622	0.6	15,525	1.6	6355	5.6	2899	13.4	1239	19.5
**C: BMI≥25 kg/m**^**2**^												
*H*. *pylori* positive	3559	1.8	11,645	4.2	12,787	10.5	7268	22.7	3839	31.6	1464	35.6
*H*. *pylori* negative	3146	1.3	8443	3.8	8941	10.3	7277	21.7	4840	31.1	2337	36.8
Gastric ulcer	103	1.0	312	5.8	432	14.1	473	27.9	315	38.4	185	38.9
No gastric ulcer	6602	1.6	19,776	4.0	21,296	10.4	14,072	22.0	8364	31.0	3616	36.2
Duodenal ulcer	271	2.6	1126	5.2	1577	11.4	1652	25.0	1050	35.0	557	38.4
No duodenal ulcer	6434	1.5	18,962	3.9	20,151	10.4	12,893	21.8	7629	30.8	3244	36.0

BMI: Body mass index; DM: diabetes mellitus

Adjustment for age showed increased likelihood for DM in relation to *H*. *pylori* infection [age-adjusted OR_MH_ 1.10 (95% CI 1.05–1.14)], gastric ulcer [age-adjusted OR_MH_ 1.31 (95% CI 1.18–1.46)] and duodenal ulcer [age-adjusted OR_MH_ 1.25 (95% CI 1.17–1.33)] (Table 3A).

**Table 3:** Age adjusted and age specific odds ratios and 95% confidence intervals of the associations of H. pylori infection, and gastric and duodenal ulcers, with diabetes mellitus, by age (in years) and BMI (< and >25 kg/m2).

	Pooled age groups	Age 25–29	Age 30–39	Age 40–49	Age 50–59	Age 60–69	Age 70–95	P for age heterogeneity
	OR_MH_ (95% CI)^a^	OR (95% CI)	OR (95% CI)	OR (95% CI)	OR (95% CI)	OR (95% CI)	OR (95% CI)	
**A: all BMI categories**								
*H*. *pylori* positive	1.10 (1.05–1.14)^b^	1.51 (1.08–2.13)	1.21 (1.06–1.38)	1.10 (1.02–1.20)	1.13 (1.05–1.21)	1.05 (0.97–1.14)	0.95 (0.84–1.07)	0.02
Gastric ulcer	1.31 (1.18–1.46)^c^	0.50 (0.08–3.97)	1.46 (0.93–2.29)	1.38 (1.07–1.77)	1.38 (1.14–1.66)	1.34 (1.10–1.65)	1.08 (0.83–1.40)	0.5
Duodenal ulcer	1.25 (1.17–1.33)^d^	2.47 (1.33–4.60)	1.43 (1.11–1.85)	1.34 (1.16–1.54)	1.25 (1.12–1.39)	1.21 (1.07–1.36)	1.13 (0.96–1.32)	0.13
**B: BMI<25 kg/m2**								
*H*. *pylori* positive	1.16 (1.05–1.29)	1.19 (0.58–2.43)	1.16 (0.83–1.60)	1.28 (1.01–1.62)	1.32 (1.08–1.61)	1.07 (0.87–1.31)	0.96 (0.73–1.25)	0.4
Gastric ulcer	1.10 (0.82–1.46)	0.99 (0.99–1.00)	0.97 (0.24–3.92)	1.19 (0.56–2.54)	0.97 (0.52–1.79)	1.36 (0.85–2.18)	0.91 (0.49–1.68)	0.8
Duodenal ulcer	1.38 (1.17–1.61)	3.62 (1.10–11.99)	1.10 (0.51–2.36)	2.53 (1.80–3.57)	1.31 (0.97–1.78)	1.17 (0.87–1.58)	1.11 (0.77–1.61)	0.004
**C: BMI≥25 kg/m2**								
*H*. *pylori* positive	1.04 (1.00–1.09)	1.49 (0.93–2.06)	1.11 (0.96–1.28)	1.02 (0.94–1.12)	1.06 (0.98–1.15)	1.03 (0.94–1.12)	0.95 (0.83–1.09)	0.29
Gastric ulcer	1.34 (1.19–1.51)	0.61 (0.09–4.43)	1.48 (0.92–2.40)	1.42 (1.08–1.87)	1.37 (1.12–1.68)	1.39 (1.10–1.75)	1.12 (0.83–1.52)	0.7
Duodenal ulcer	1.18 (1.10–1.27)	1.71 (0.79–3.73)	1.35 (1.03–1.78)	1.11 (0.94–1.30)	1.19 (1.06–1.34)	1.21 (1.06–1.39)	1.11 (0.92–1.34)	0.7

BMI: Body mass index; CI: confidence intervals; OR: Odds ratio

^a^ Age-adjusted OR by Mantel-Haenzel. Each independent variable (*H*. *pylori* infection, gastric ulcer and duodenal ulcer) was analyzed separately

^b^ P = 0.05,

^c^ P = 0.2,

^d^ P = 0.08 by chi square test for heterogeneity across BMI categories.

There was evidence (P = 0.02 for heterogeneity) for a positive association between *H*. *pylori* infection and DM prevalence, which differed by age: OR range of 1.51–1.10 for ages 25–59 years, and non-significant associations for ages 60–95 years (Table 3). The association between gastric ulcer and DM prevalence was statistically significant for ages 40–69, although the test for heterogeneity was not statistically significant (P = 0.5). The strength of the association of duodenal ulcer with DM prevalence differed slightly by age group (P = 0.13 by chi square test for heterogeneity) (Table 3A).

The heterogeneity test showed borderline statistical significance when pooling the age-adjusted OR_MH_ of the associations of *H*. *pylori* infection and duodenal ulcer with DM across BMI categories (P = 0.05 and P = 0.08, respectively) (Table 3A–3C).

#### Multivariable analysis

In pooled multivariable analyses, the positive associations of *H*. *pylori* infection, gastric ulcer and duodenal ulcer, with DM, were slightly attenuated but remained significant: aOR 1.05 (95% CI 1.05–1.10), 1.20 (95% CI 1.06–1.34) and 1.20 (95% CI 1.12–1.28), respectively, after adjustment for age, socioeconomic status of place of residence, country of birth, smoking, BMI, dyslipidemia and hypertension. The results were similar when including into the same model the variables *H*. *pylori* positivity (by UBT) and gastric ulcer or duodenal ulcer: aOR 1.07 (95% CI 1.03–1.12), 1.20 (95% CI 1.06–1.33), 1.20 (95% CI 1.11–1.27) respectively.

Multivariable adjusted associations of *H*. *pylori* infection, gastric ulcer and duodenal ulcer, with DM, by age and BMI groups, differed slightly across the sub-groups. However, no significant heterogeneity by age group was found. The adjusted association of *H*. *pylori* infection with DM was stronger in people with BMI<25 kg/m^2^ than in those with BMI ≥25 kg/m^2^ (P = 0.04 by chi square test for heterogeneity); however, for the adjusted associations of gastric ulcer and duodenal ulcer with DM, no significant heterogeneity by BMI was found (P = 0.3) (Table 4A–4C).

**Table 4:** Adjusted odds ratio and 95% confidence intervals of the associations of H. pylori infection, and gastric and duodenal ulcers, with diabetes mellitus by age (in years) and BMI (< and >25 kg/m2).

	Pooled age groups	Age 25–29	Age 30–39	Age 40–49	Age 50–59	Age 60–69	Age 70–95	P for age heterogeneity
	aOR (95% CI)	aOR (95% CI)	aOR (95% CI)	aOR (95% CI)	aOR (95% CI)	aOR (95% CI)	aOR (95% CI)	
**A: all BMI categories** ^a^								
*H*. *pylori* positive	**1.05 (1.01–1.10)** ^c^	**1.43 (1.00–2.05)**	1.11 (0.96–1.27)	1.03 (0.94–1.12)	1.09 (1.01–1.18)	1.03 (0.95–1.13)	0.94 (0.83–1.06)	0.16
Gastric ulcer	**1.20 (1.06–1.34)** ^d^	0.51 (0.06–3.33)	1.19 (0.91–1.55)	1.19 (0.90–1.56)	**1.20 (0.98–1.48)**	**1.28 (1.03–1.59)**	1.08 (0.81–1.43)	0.9
Duodenal ulcer	**1.20 (1.12–1.28)** ^e^	**1.96 (0.99–3.87)**	1.18 (0.73–1.89)	**1.18 (1.00–1.38)**	**1.20 (1.06–1.34)**	**1.22 (1.07–1.39)**	1.09 (0.92–1.30)	0.6
**B: BMI<25 kg/m**^**2**^ ^b^								
*H*. *pylori* positive	**1.16 (1.04–1.29)**	1.18 (0.57–2.42)	1.10 (0.79–1.53)	1.21 (0.95–1.54)	**1.35 (1.10–1.66)**	1.05 (0.85–1.29)	0.99 (0.75–1.30)	0.4
Gastric ulcer	1.06 (0.79–1.42)	NA	0.81 (0.20–3.35)	0.96 (0.44–2.06)	0.88 (0.47–1.66)	1.30 (0.80–2.11)	1.01 (0.54–1.90)	0.8
Duodenal ulcer	**1.27 (1.07–1.49)**	**3.26 (0.98–10.90)**	0.93 (0.43–2.01)	**2.10 (1.47–3.00)**	1.20 (0.84–1.59)	1.16 (0.86–1.58)	1.06 (0.72–1.55)	0.038
**C: BMI≥25 kg/m**^**2**^ ^a^								
*H*. *pylori* positive	1.03 (0.98–1.08)	**1.57 (1.03–2.37)**	1.11 (0.95–1.29)	1.00 (0.91–1.10)	1.05 (0.97–1.14)	1.03 (0.94–1.14)	0.93 (0.81–1.07)	0.18
Gastric ulcer	**1.22 (1.08–1.39)**	0.51 (0.07–3.80)	1.24 (0.75–2.06)	1.23 (0.92–1.65)	**1.25 (1.01–1.56)**	**1.23 (1.07–1.42)**	1.09 (0.79–1.49)	0.9
Duodenal ulcer	**1.17 (1.09–1.26)**	1.61 (0.71–3.63)	1.22 (0.92–1.63)	1.05 (0.88–1.24)	**1.20 (1.04–1.34)**	**1.28 (1.01–1.64)**	1.10 (0.90–1.33)	0.6

aOR: adjusted odds ratio; BMI: Body mass index; CI: confidence intervals; NA: not applicable, no cases of gastric ulcer in patients aged 25–29 years. Multivariable models presented in this table were fitted separately for each independent variable: *H*. *pylori* infection (by UBT), gastric ulcer and duodenal ulcer.

^a^ Adjusted for age, socioeconomic status (SES) of place of residence, country of birth, BMI, smoking, dyslipidemia and hypertension.

^b^ Adjusted for age, SES of place of residence, country of birth, smoking, dyslipidemia and hypertension. The age specific models did not include age.

^c^ P = 0.049,

^d^ P = 0.3,

^e^ P = 0.3 by chi square test for heterogeneity across BMI categories.

An association between gastric ulcer and DM was found in both *H*. *pylori* negative persons aOR 1.24 (95% CI 1.06–1.44) and *H*. *pylori* positive persons: aOR 1.17 (95% CI 0.98–1.40); P = 0.6 by chi square test for heterogeneity. Similarly, an association between duodenal ulcer and DM was found in both *H*. *pylori* negative aOR 1.20 (95% CI 1.10–1.31) and positive persons: aOR 1.25 (95% CI 1.12–1.39); P = 0.5 by chi square test for heterogeneity.

## Discussion

In this large population-based study, we assessed associations of *H*. *pylori* infection and its related gastroduodenal diseases with DM, among adults aged 25–95 years. To our knowledge, this is the largest study that addressed the role of *H*. *pylori* infection in DM. The overall association of *H*. *pylori* infection (by UBT) with DM was close to null (aOR 1.05 (95% CI 1.01–1.10)), although this association was stronger in patients with BMI<25 kg/m^2^ (aOR 1.17 (95% CI 1.04–1.29)), suggesting an effect modification by BMI (P for heterogeneity 0.049). We found significant positive associations of gastric and duodenal ulcers with DM, independent of known risk factors for DM such as age, socioeconomic status, BMI, dyslipidemia, hypertension and smoking. These associations were of small magnitude (aOR 1.20 (95% CI 1.06–1.34) and 1.20 (95% CI 1.12–1.28), respectively).

Previous studies that addressed the association between *H*. *pylori* infection and DM reported conflicting results. Some demonstrated a significant positive association between *H*. *pylori* infection and DM [8, 16, 26–28], while others reported null results [14, 17, 29] or positive associations that became non-significant following adjustment for potential confounders [9, 13, 30, 31]. These discrepancies might be explained, at least in part, by different methods employed across the studies in the detection of *H*. *pylori* infection, and in sample size and/or selection of the study population. Also, these studies employed various definitions of DM, some followed the American Diabetes Association criteria [14, 16, 28]. Others used one of several criteria such as reports on anti-diabetic medications, self-report physician diagnosis of DM or fasting blood glucose [8, 9, 15].

Our findings indicate complex patterns of associations between *H*. *pylori* infection and DM, and between peptic diseases and DM. First, the positive associations of gastric and duodenal ulcers with DM were attenuated when controlling for known risk factors for DM such as age, socioeconomic status, BMI, dyslipidemia, hypertension and smoking. The associations did not completely diminish; rather, adjustment had limited impact on the strength of the associations. This suggests that these factors may comprise partial confounders in the association between peptic ulcer disease and DM.

Second, we report a possible effect modification by BMI. The association between *H*. *pylori* infection and DM became more evident in people with BMI<25 kg/m^2^; although some variation in the association measure was observed across the BMI/age groups. Testing for heterogeneity by age was mostly not significant. Previous studies also proposed that *H*. *pylori* infection and DM might be modified by age and BMI [28, 32]. These observations highlight the importance of large population-based studies for examining an independent role of *H*. *pylori* infection, beyond well-known and established risk factors for DM.

The current evidence does not enable determination of causality in the observed associations of *H*. *pylori* infection, and gastric and duodenal ulcers, with DM. *H*. *pylori* infection might be a marker for unmeasured characteristics. Nonetheless, whether *H*. *pylori* is causally related to DM or is simply a risk marker, our observations have public health and clinical importance, and suggest that patients infected with *H*. *pylori* and those with peptic disease warrant special attention regarding the prevention of DM.

*H*. *pylori* infection is generally acquired in early childhood [33], several years before the usual onset of DM. *H*. *pylori* gastric colonization induces rigorous humoral and cell-mediated immune responses [10, 34], which do not clear the infection. The inflammation induced by *H*. *pylori* might provide a possible explanation for the positive associations observed of *H*. *pylori* infection and peptic disease with DM. The predominant human T cell response is the T-helper 1 mediated response, which is associated with releasing proinflammatory cytokines and activation of phagocytes [10, 34, 35]. *H*. *pylori* also induces Th2 and T-regulatory (Tregs) responses [10, 34, 35]. Imbalance between Th1 and Tregs responses might induce peptic disease [34, 36]. Our finding of a positive association between peptic ulcer disease and DM suggests that the long-term gastric inflammation and induced-damage to the gastric mucosa are likely involved in the relationship between *H*. *pylori* infection and DM.

*H*. *pylori*-induced inflammation affects gastric physiology. For example, *H*. *pylori* affects the levels of pepsinogen (PG) I and PGII; proenzymes of the digestive enzyme pepsin. PGI and PGII are secreted from cells in the corpus and PGII is also secreted from cells in the antrum and duodenum [37, 38]. Serum PGI and PGII are increased in *H*. *pylori* infected vs. uninfected individuals, and higher levels are found in those with more severe gastritis [39]. As the severity of gastritis progresses and corpus atrophic lesions appear, the PGI level decreases, while the PGII level remains stable; the result is a decrease in the PGI:PGII ratio [39, 40]. These markers predict various gastric pathologies [39]. A recent study [41] showed significant negative correlations between the PGI:PGII ratio and cardiovascular risk factors among individuals with type 2 DM [41]. *H*. *pylori* infection can also affect the regulation of ghrelin and leptin [42–44], two hormones that have important roles in energy homeostasis [45]; both hormones are secreted by the epithelial cells in the stomach [45, 46]. Ghrelin decreases energy expenditure and stimulates weight gain [47], while leptin reduces appetite and increases energy expenditure [45]. Altogether, these studies suggest that *H*. *pylori* can alter gastric physiology, which can in turn affect metabolic homeostasis and the risk of DM.

In the current study, *H*. *pylori* infections were detected in 52.0% of individuals who performed UBT. This is comparable with previous data on *H*. *pylori* positivity among Israeli adults, as well as the variation observed according to country of birth [21]. However, unexpectedly, the prevalence of the infection (by UBT) was lower in the subgroups of persons aged 50–95 years than in the younger age subgroups; this observation was not explained by country of birth, and suggests possible differences in referrals to UBT by age group. The prevalence of DM (8.3%) is also comparable to previous estimates (8.3–9.5%), despite the use of different criteria to determine DM [48, 49].

Our study has some limitations. We used data from a large HMO database, which were collected primarily for administrative purposes. The methods of collecting information on variables such as BMI and smoking may vary among medical personnel within the same HMO. Missing information was low for BMI, country of birth and socioeconomic status; yet relatively high (27%) for smoking. In persons with unknown smoking status, the prevalence of *H*. *pylori* infection was similar to that in never smokers, and DM prevalence was lower compared to those with documented smoking status. This might have resulted in partial adjustment for smoking.

Our study also has several strengths. First is the use of a large population-base sample. Second is the employment of standard criteria for the classification of DM, which combine results of laboratory tests, purchases of anti-diabetic medications and physicians' diagnoses. Lastly, *H*. *pylori* infection was determined based on UBT, which is among the most accurate non-invasive methods for the detection of *H*. *pylori*. Since only physicians can refer patients to UBT, our study population most likely represents symptomatic persons.

### Conclusions

Significant positive associations of small magnitude were found between *H*. *pylori* infection and DM, and between peptic disease and DM, independent of known determinants of DM. The long-term gastric inflammation and associated-damage to the gastric mucosa, as reflected by peptic disease, might play a role in the association between *H*. *pylori* infection and DM. Further studies are needed to elucidate the underlying mechanisms of these associations.

## Supporting information

S1 Table Correlates of diabetes mellitus. (DOCX)

## References

[1] van Dieren S, Beulens JW, van der Schouw YT, Grobbee DE, Neal B. The global burden of diabetes and its complications: an emerging pandemic. Eur J Cardiovasc Prev Rehabil. 2010;17 Suppl 1:S3–8. doi: 10.1097/01.hjr.0000368191.86614.5a 20489418 10.1097/01.hjr.0000368191.86614.5a

[2] Powers AC. Harrison's Principles of Internal Medicine, 18e 18e ed Chapter 344. Diabetes Mellitus. The McGraw-Hill Companies, Inc.; 2013.

[3] Medalie JH, Papier CM, Goldbourt U, Herman JB. Major factors in the development of diabetes mellitus in 10,000 men. Arch Intern Med. 1975;135(6):811–7. 1130926

[4] Espelt A, Arriola L, Borrell C, Larranaga I, Sandin M, Escolar-Pujolar A. Socioeconomic position and type 2 diabetes mellitus in Europe 1999–2009: a panorama of inequalities. Curr Diabetes Rev. 2011;7(3):148–58. 21418003 10.2174/157339911795843131

[5] Agardh E, Allebeck P, Hallqvist J, Moradi T, Sidorchuk A. Type 2 diabetes incidence and socio-economic position: a systematic review and meta-analysis. Int J Epidemiol. 2011;40(3):804–18. doi: 10.1093/ije/dyr029 21335614 10.1093/ije/dyr029

[6] Steyn NP, Mann J, Bennett PH, Temple N, Zimmet P, Tuomilehto J et al Diet, nutrition and the prevention of type 2 diabetes. Public Health Nutr. 2004;7(1A):147–65. 14972058 10.1079/phn2003586

[7] Hu FB, Manson JE, Stampfer MJ, Colditz G, Liu S, Solomon CG et al Diet, lifestyle, and the risk of type 2 diabetes mellitus in women. N Engl J Med. 2001;345(11):790–7. doi: 10.1056/NEJMoa010492 11556298 10.1056/NEJMoa010492

[8] Jeon CY, Haan MN, Cheng C, Clayton ER, Mayeda ER, Miller JW et al Helicobacter pylori infection is associated with an increased rate of diabetes. Diabetes Care. 2012;35(3):520–5. doi: 10.2337/dc11-1043 22279028 10.2337/dc11-1043 PMC3322696

[9] Lutsey PL, Pankow JS, Bertoni AG, Szklo M, Folsom AR. Serological evidence of infections and Type 2 diabetes: the MultiEthnic Study of Atherosclerosis. Diabet Med. 2009;26(2):149–52. doi: 10.1111/j.1464-5491.2008.02632.x 19236617 10.1111/j.1464-5491.2008.02632.x PMC2679689

[10] Atherton JC. The pathogenesis of Helicobacter pylori-induced gastro-duodenal diseases. Annu Rev Pathol. 2006;1:63–96. doi: 10.1146/annurev.pathol.1.110304.100125 18039108 10.1146/annurev.pathol.1.110304.100125

[11] Baudron CR, Franceschi F, Salles N, Gasbarrini A. Extragastric diseases and Helicobacter pylori. Helicobacter. 2013;18:44–51. doi: 10.1111/hel.12077 24011245 10.1111/hel.12077

[12] Malfertheiner P, Megraud F, O'Morain CA, Atherton J, Axon ATR, Bazzoli F et al Management of Helicobacter pylori infection-the Maastricht IV/ Florence Consensus Report. Gut. 2012;61(5):646–64. doi: 10.1136/gutjnl-2012-302084 22491499 10.1136/gutjnl-2012-302084

[13] Dore MP, Bilotta M, Malaty HM, Pacifico A, Maioli M, Graham DY et al Diabetes mellitus and Helicobacter pylori infection. Nutrition. 2000;16(6):407–10. 10869894 10.1016/s0899-9007(00)00267-7

[14] Demir M, Gokturk HS, Ozturk NA, Kulaksizoglu M, Serin E, Yilmaz U. Helicobacter pylori prevalence in diabetes mellitus patients with dyspeptic symptoms and its relationship to glycemic control and late complications. Dig Dis Sci. 2008;53(10):2646–9. doi: 10.1007/s10620-007-0185-7 18320319 10.1007/s10620-007-0185-7

[15] Chen Y, Blaser MJ. Association between gastric Helicobacter pylori colonization and glycated hemoglobin levels. J Infect Dis. 2012;205(8):1195–202. doi: 10.1093/infdis/jis106 22427676 10.1093/infdis/jis106 PMC3308905

[16] Bener A, Micallef R, Afifi M, Derbala M, Al-Mulla HM, Usmani MA. Association between type 2 diabetes mellitus and Helicobacter pylori infection. Turk J Gastroenterol. 2007;18(4):225–9. 18080918

[17] Xia HHX, Talley NJ, Kam EPY, Young LJ, Hammer J, Horowitz M. Helicobacter pylori infection is not associated with diabetes mellitus, nor with upper gastrointestinal symptoms in diabetes mellitus. Am J Gastroenterol. 2001;96(4):1039–46. 11316144 10.1111/j.1572-0241.2001.03604.x

[18] Zhou X, Zhang C, Wu J, Zhang G. Association between Helicobacter pylori infection and diabetes mellitus: a meta-analysis of observational studies. Diabetes Res Clin Pract. 2013;99(2):200–8. doi: 10.1016/j.diabres.2012.11.012 23395214 10.1016/j.diabres.2012.11.012

[19] Wang F, Liu J, Lv ZS. Association of Helicobacter pylori infection with diabetes mellitus and diabetic nephropathy: A meta-analysis of 39 studies involving more than 20,000 participants. Scand J Infect Dis. 2013;45(12):930–8. doi: 10.3109/00365548.2013.844351 24143873 10.3109/00365548.2013.844351

[20] Harris MI. Epidemiologic studies on the pathogenesis of non-insulin-dependent diabetes mellitus (NIDDM). Clin Invest Med. 1995;18(4):231–9. 8549007

[21] Muhsen K, Cohen D, Spungin-Bialik A, Shohat T. Seroprevalence, correlates and trends of Helicobacter pylori infection in the Israeli population. Epidemiol Infect. 2012;140(7):1207–14. doi: 10.1017/S0950268811002081 22014090 10.1017/S0950268811002081

[22] Heymann AD, Chodick G, Halkin H, Karasik A, Shalev V, Shemer J et al The implementation of managed care for diabetes using medical informatics in a large Preferred Provider Organization. Diabetes Res Clin Pr. 2006;71(3):290–8. doi: 10.1016/j.diabres.2005.07.002 16112245 10.1016/j.diabres.2005.07.002

[23] Weitzman D, Chodick G, Shalev V, Grossman C, Grossman E. Prevalence and factors associated with resistant hypertension in a large health maintenance organization in Israel. Hypertension. 2014;64(3):501–7. doi: 10.1161/HYPERTENSIONAHA.114.03718 24958503 10.1161/HYPERTENSIONAHA.114.03718

[24] Israel Centeral Bureau of Statistics. Characterization and classification of geagraphic units by the socio-economic level of the population 2008. 2013.

[25] Abramson JH. WINPEPI updated: computer programs for epidemiologists, and their teaching potential. Epidemiol Perspect Innov. 2011;8(1):1 doi: 10.1186/1742-5573-8-1 21288353 10.1186/1742-5573-8-1 PMC3041648

[26] Devrajani BR, Shah SZ, Soomro AA, Devrajani T. Type 2 diabetes mellitus: A risk factor for Helicobacter pylori infection: A hospital based case-control study. Int J Diabetes Dev Ctries. 2010;30(1):22–6. 20431802 10.4103/0973-3930.60008 PMC2859280

[27] Bajaj S, Rekwal L, Misra SP, Misra V, Yadav RK, Srivastava A. Association of helicobacter pylori infection with type 2 diabetes. Indian J Endocrinol Metab. 2014;18(5):694–9. 25285288 10.4103/2230-8210.139235 PMC4171894

[28] Hsieh MC, Wang SS, Hsieh YT, Kuo FC, Soon MS, Wu DC. Helicobacter pylori infection associated with high HbA1c and type 2 diabetes. Eur J Clin Invest. 2013;43(9):949–56. doi: 10.1111/eci.12124 23879740 10.1111/eci.12124

[29] Ko GT, Chan FK, Chan WB, Sung JJ, Tsoi CL, To KF et al Helicobacter pylori infection in Chinese subjects with type 2 diabetes. Endocr Res. 2001;27(1–2):171–7. 11428708 10.1081/erc-100107178

[30] Gillum RF. Infection with Helicobacter pylori, coronary heart disease, cardiovascular risk factors, and systemic inflammation: the Third National Health and Nutrition Examination Survey. J Natl Med Assoc. 2004;96(11):1470–6. 15586651 PMC2568603

[31] Tamura T, Morita E, Kawai S, Sasakabe T, Sugimoto Y, Fukuda N et al No association between Helicobacter pylori infection and diabetes mellitus among a general Japanese population: a cross-sectional study. Springerplus. 2015;4 Artn 602 doi: 10.1186/S40064-015-1371-2 10.1186/s40064-015-1371-2 PMC4627969 26543737

[32] Han X, Li Y, Wang J, Liu B, Hu H, Li X et al Helicobacter pylori infection is associated with type 2 diabetes among a middle- and old-age Chinese population. Diabetes Metab Res Rev. 2016;32(1):95–101. doi: 10.1002/dmrr.2677 26172433 10.1002/dmrr.2677

[33] Muhsen K, Jurban M, Goren S, Cohen D. Incidence, age of acquisition and risk factors of Helicobacter pylori infection among Israeli Arab infants. J Trop Pediatr. 2012;58(3):208–13. doi: 10.1093/tropej/fmr068 21908868 10.1093/tropej/fmr068

[34] Atherton JC, Blaser MJ. Coadaptation of Helicobacter pylori and humans: ancient history, modern implications. J Clin Invest. 2009;119(9):2475–87. doi: 10.1172/JCI38605 19729845 10.1172/JCI38605 PMC2735910

[35] Goll R, Gruber F, Olsen T, Cui G, Raschpichler G, Buset M et al Helicobacter pylori stimulates a mixed adaptive immune response with a strong T-regulatory component in human gastric mucosa. Helicobacter. 2007;12(3):185–92. doi: 10.1111/j.1523-5378.2007.00495.x 17492997 10.1111/j.1523-5378.2007.00495.x

[36] Robinson K, Kenefeck R, Pidgeon EL, Shakib S, Patel S, Polson RJ et al Helicobacter pylori-induced peptic ulcer disease is associated with inadequate regulatory T cell responses. Gut. 2008;57(10):1375–85. doi: 10.1136/gut.2007.137539 18467372 10.1136/gut.2007.137539

[37] Samloff IM. Cellular localization of group I pepsinogens in human gastric mucosa by immunofluorescence. Gastroenterology. 1971;61(2):185–8. 4935210

[38] Samloff IM, Liebman WM. Cellular localization of the group II pepsinogens in human stomach and duodenum by immunofluorescence. Gastroenterology. 1973;65(1):36–42. 4124404

[39] Miki K, Urita Y. Using serum pepsinogens wisely in a clinical practice. J Dig Dis. 2007;8(1):8–14. doi: 10.1111/j.1443-9573.2007.00278.x 17261129 10.1111/j.1443-9573.2007.00278.x

[40] Graham DY, Nurgalieva ZZ, El-Zimaity HM, Opekun AR, Campos A, Guerrero L et al Noninvasive versus histologic detection of gastric atrophy in a Hispanic population in North America. Clin Gastroenterol Hepatol. 2006;4(3):306–14. doi: 10.1016/j.cgh.2005.11.003 16527693 10.1016/j.cgh.2005.11.003

[41] Bahadoran Z, Mirmiran P, Zarif-Yeaganeh M, Zojaji H, Azizi F. Helicobacter pylori Stool Antigen Levels and Serological Biomarkers of Gastric Inflammation are Associated with Cardio-Metabolic Risk Factors in Type 2 Diabetic Patients. Endocrinol Metab. 2015;30(3):280–7. doi: 10.3803/EnM.2015.30.3.280 26435133 10.3803/EnM.2015.30.3.280 PMC4595352

[42] Roper J, Francois F, Shue PL, Mourad MS, Pei Z, Olivares de Perez AZ et al Leptin and ghrelin in relation to Helicobacter pylori status in adult males. Clin Endocrinol Metab. 2008;93(6):2350–7. doi: 10.1210/jc.2007-2057 18397989 10.1210/jc.2007-2057 PMC2435636

[43] Francois F, Roper J, Joseph N, Pei Z, Chhada A, Shak JR et al The effect of H. pylori eradication on meal-associated changes in plasma ghrelin and leptin. BMC Gastroenterology. 2011;11:37 doi: 10.1186/1471-230X-11-37 21489301 10.1186/1471-230X-11-37 PMC3089783

[44] Chuang CH, Sheu BS, Yang HB, Lee SC, Kao AW, Cheng HC et al Gender difference of circulating ghrelin and leptin concentrations in chronic Helicobacter pylori infection. Helicobacter. 2009;14(1):54–60. doi: 10.1111/j.1523-5378.2009.00653.x 19191897 10.1111/j.1523-5378.2009.00653.x

[45] Cummings DE, Overduin J. Gastrointestinal regulation of food intake. J Clin Invest. 2007;117(1):13–23. doi: 10.1172/JCI30227 17200702 10.1172/JCI30227 PMC1716217

[46] Bado A, Levasseur S, Attoub S, Kermorgant S, Laigneau JP, Bortoluzzi MN et al The stomach is a source of leptin. Nature. 1998;394(6695):790–3. doi: 10.1038/29547 9723619 10.1038/29547

[47] Kojima M, Hosoda H, Date Y, Nakazato M, Matsuo H, Kangawa K. Ghrelin is a growth-hormone-releasing acylated peptide from stomach. Nature. 1999;402(6762):656–60. doi: 10.1038/45230 10604470 10.1038/45230

[48] Israel Minsitry of Health. National program for quality indicators in community health care. 2014.

[49] Israel Minsitry of Health. Israel national health interview survey 2007–2010; selected findings. Israel Center for Disease Control. Tal Hashomer 2012.

