# Supplementary material for: Associations of Helicobacter pylori infection and peptic disease with diabetic mellitus: Results from a large population-based study
Source: PLoS One. 2017 Aug 29;12(8):e0183687. doi: 10.1371/journal.pone.0183687 (PMC5574557; doi:10.1371/journal.pone.0183687)
Supplement: S1 Table — (DOCX) [file pone.0183687.s001.docx]

**S1 Table. Correlates of diabetes mellitus**

|  | **Total** | **Diabetes mellitus, N (%)** |
| --- | --- | --- |
| **Age (years)** |  |  |
| 25-34 | 45,054 | 549 (1.0%) |
| 35-64 | 92,641 | 8655 (9.3%) |
| ≥65 | 10,241 | 3103 (30.3%) |
| **Sex** |  |  |
| Men | 58,173 | 5773 (9.9%) |
| Women | 89,763 | 6434 (7.2%) |
| **SES rank ^a^** |  |  |
| 1-5 | 60,282 | 5694 (9.4%) |
| 6-7 | 39,500 | 3226 (8.2%) |
| 8-10 | 38,790 | 2719 (7.0%) |
| Missing | 9364 | 568 (6.1%) |
| **Country of Birth** |  |  |
| Israel | 96,180 | 6276 (6.5%) |
| Former Soviet Union countries | 35,885 | 3497 (9.7%) |
| Asia/ North Africa | 5379 | 1004 (18.7%) |
| Europe ^b^ /America | 7009 | 942 (13.4%) |
| Other/unknown | 3483 | 488 (14.0%) |
| **BMI (kg/m^2^) ^c^** |  |  |
| <18.5 | 4139 | 20 (0.5%) |
| 18.5-24 | 62,053 | 1587 (2.6%) |
| 25-29 | 49,636 | 4741 (9.6%) |
| ≥30 | 25,910 | 5762 (22.2%) |
| Missing | 6198 | 97 (1.6%) |
| **Smoking** |  |  |
| Ever | 20,913 | 1869 (8.9%) |
| Never | 86,540 | 7333 (8.5%) |
| Unknown | 40,483 | 3005 (7.4%) |
| **Hypertension** | 33,317 | 8159 (24.5%) |
| No hypertension | 114,619 | 4048 (3.5%) |
| **Dyslipidemia** | 46,637 | 8949 (19.2%) |
| No dyslipidemia | 101,299 | 3258 (3.2%) |

^a^ Socioeconomic status (SES) rank of city/town of residence; 1-5 represents low SES; 6-7, intermediate SES; and 8-10, high SES.

^b^ Excluding countries of the Former Soviet Union.

^c^ BMI: body mass index; kg: kilogram; m: meters
